# Lung flukes of the genus *Paragonimus*: ancient and re-emerging pathogens

**DOI:** 10.1017/S0031182022000300

**Published:** 2022-09

**Authors:** David Blair

**Affiliations:** College of Science and Engineering, James Cook University, Townsville, Queensland 4811, Australia

**Keywords:** Evolution, foodborne trematodes, genomics, life cycle, lung flukes, molecular taxonomy, paragonimiasis, *Paragonimus*

## Abstract

The title of this article refers to Table 1 in Zhou (2022, Infectious diseases of poverty: progress achieved during the decade gone and perspectives for the future. Infectious Diseases of Poverty 11, 1), in which it is indicated that *Paragonimus* species, like many other foodborne trematodes, are ancient pathogens that are also re-emerging to cause disease in modern times. This article provides a general overview of *Paragonimus* species and the disease they cause. This is followed by comments on several specific topics of current interest: taxonomy and distribution of members of the genus; details of the life cycle; global and regional prevalence of paragonimiasis; genomics of lung flukes and possible effects of global environmental change. Unresolved questions relating to these topics are discussed and gaps in knowledge identified.

## Graphical abstract

**Figure:**
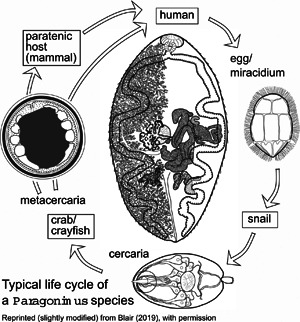

## Introduction

Foodborne trematodes target their hosts, including humans, by residing in tasty food items. Lung flukes of the genus *Paragonimus* are no exception. Humans like to eat crabs and crayfish, often raw or incompletely cooked. If these crustaceans harbour metacercariae of a *Paragonimus* species, then human infection (paragonimiasis) can ensue. Paragonimiasis is unusual among foodborne zoonotic trematodes in having a very broad geographical distribution (tropical and some subtropical regions of Asia, Africa and the Americas), a large number of actual or potential causative *Paragonimus* species and a considerable range of clinical signs and symptoms, many of which mimic those of other diseases, especially tuberculosis and cancer. The disease is also unusual in that infection, although usually acquired by eating freshwater crustaceans containing metacercariae, can also arise following ingestion of uncooked meat of mammalian paratenic hosts.

There are several recent general reviews on paragonimiasis and the genus *Paragonimus* that cover many aspects of their biology, evolution and medical significance. These include Adams (2018), Doanh *et al*. (2018*a*), Blair (2019) and Chai and Jung (2019) (all general reviews); Yoshida *et al*. (2019) (Asia); Zhou *et al*. (2021) (China); Cumberlidge *et al*. (2018) and Rabone *et al*. (2021) (both focused on Africa); Tenorio and Molina (2021) (Philippines); Coogle *et al*. (2021) (North America); Calvopiña *et al*. (2014) (Ecuador). The Centers for Disease Control website https://www.cdc.gov/parasites/paragonimus/ gives online information for those who prefer that format. Other reviews on specific aspects are cited later.

This article will focus on only a few topics, updating these and pointing out where further investigation is required. But first, an introduction is required to paragonimiasis and the trematodes that cause it.

**Fig. 1.**
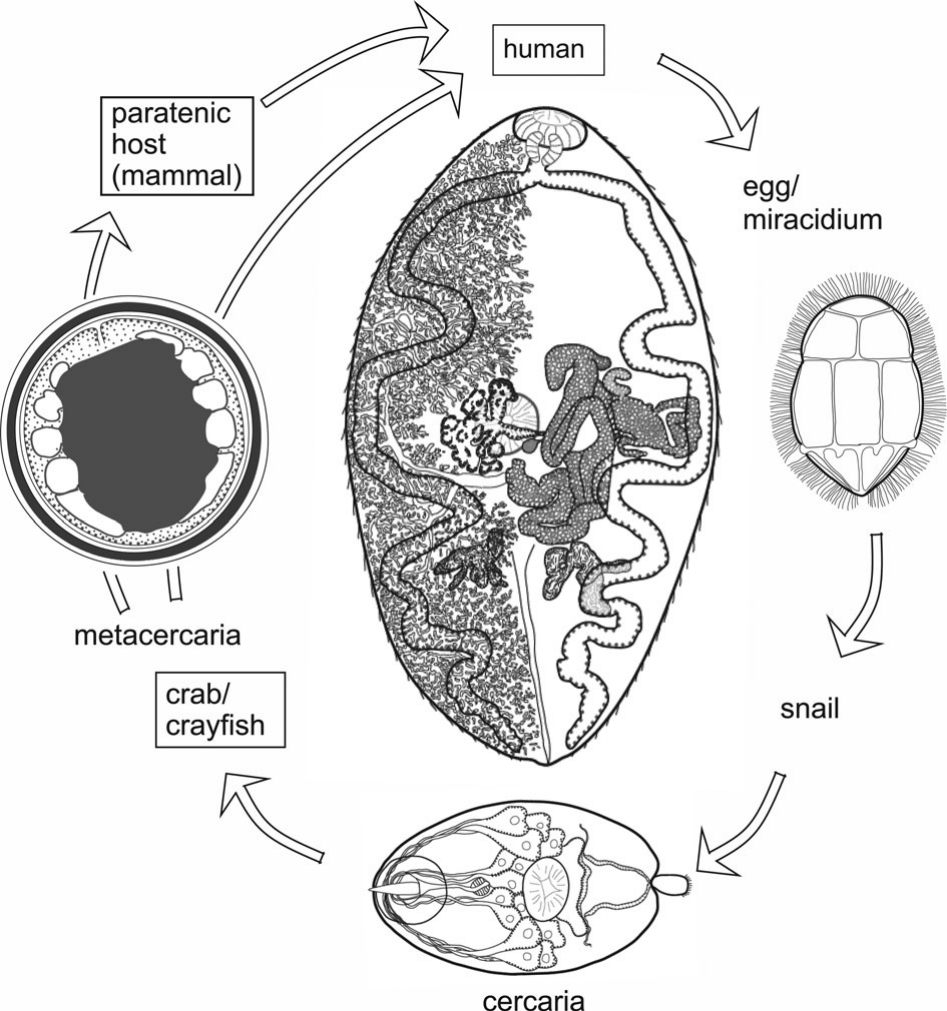
Life cycle typical of *Paragonimus* species (see text for details). Image reprinted with permission of Springer Nature Switzerland AG from Blair (2019). The drawing of the adult in the centre of the figure is a dorsal view based on a photograph of a strongly compressed specimen of *P*. *westermani* from Vietnam, supplied by Dr P. N. Doanh. Vitellaria are indicated on the left side of the body only.

The genus *Paragonimus* is found in parts of Asia, Africa and the Americas. Many different species have been named: some are zoonotic, potentially causing disease in humans. Life cycles have been worked out for some of the important species (Fig. 1) and are likely to be similar in all species. The definitive hosts are always mammals, in the lung tissue of which hermaphroditic adult worms, resembling coffee beans in size and shape, encapsulate in pairs and produce eggs. The eggs escape to the outside environment *via* sputum or faeces. In freshwater, miracidia emerging from eggs enter the haemocoel of aquatic snails and give rise eventually to cercariae. These enter crustaceans (crabs or crayfish) in which they encyst as metacercariae. Ingestion of infected crustaceans by a mammal completes the cycle. Metacercariae excyst in the gut of the mammal and penetrate the intestinal wall to migrate through tissues to the pleural cavities. Typically, individual worms seem to remain in the pleural spaces until they encounter another worm. They then enter the lung tissues and form a capsule in which the pair resides (Blair *et al*., 1999). In many lung-fluke species, paratenic hosts are optionally included in the life cycle: metacercariae in ingested crustaceans fail to develop to adulthood in animals other than specific definitive hosts, instead remaining alive in the tissues as juveniles. Rodents, deer and pigs are among the mammal groups that can act in this way. Animals other than mammals might also function as paratenic hosts (e.g. Urabe and Marcaida, 2022). Consumption of paratenic hosts by carnivorous definitive hosts completes the cycle. In this way, top predators such as tigers, which might not eat crabs directly, can become infected (Blair *et al*., 1999; Voronova *et al*., 2020), as can human hunters snacking on raw boar meat in Japan (e.g. Nakashima *et al*., 2021).

The exact manifestation of human infection depends on the species and population of *Paragonimus* involved and other factors such as the number of worms and even the ploidy of the worms (Blair, 2019). Table 1 lists the principal species that have been found in humans, and the main kinds of disease that they can cause. In natural definitive hosts, and in humans infected with certain species of *Paragonimus*, paragonimiasis is pulmonary: as mentioned above, adult worms encapsulate in pairs in the lung parenchyma and eggs pass out of these capsules into bronchioles, from which they may be voided to the outside *via* sputum or, if swallowed, *via* faeces. Mortality is low, but morbidity can be high (Feng *et al*., 2018). Symptoms of pulmonary paragonimiasis include cough, haemoptysis, chest pains, dyspnoea and weight loss (Ahn *et al*., 2021). These symptoms frequently mimic those of tuberculosis, pneumonia, lung cancer and even coronavirus disease-2019 (e.g. Gluchowska *et al*., 2021). In light infections, such as might occur when prevalence falls due to successful control campaigns, a mate may not be found – individual worms remain in the pleural spaces where they may wander around and provoke host responses leading to inflammation and conditions such as pleural effusion (Nakamura-Uchiyama *et al*., 2002). This has been demonstrated for *P. westermani* (Kerbert, 1878) but might apply to other species. A further unusual feature of *P. westermani* is that triploid forms are common in Korea, Japan and parts of China. Triploid worms produce viable eggs parthenogenetically and are physically larger than diploids. Not needing to find a mate, a single triploid worm may form a capsule in the lungs from which it voids eggs. Metacercarial cysts and eggs of triploids are also larger than those of diploids.

**Table 1.** Species of *Paragonimus* known to cause human disease, with notes their geographical distribution, nature of disease caused and natural definitive hosts

Species	Geographical distribution	Type of paragonimiasis caused in humans	Natural definitive hosts	Other notes
*P*. *westermani* complex: diploid worms	E Asia (China and Taiwan, Korea, Japan, SE Siberia); SE Asia (Philippines, Malaysia, Thailand, Cambodia, Laos, Vietnam), S Asia (Sri Lanka, India), possibly also Nepal, Pakistan and Papua New Guinea	Usually pulmonary; pleural disease not uncommon and other forms sometimes	Cercopithecids, canids, felids, herpestids, viverrids, mustelids, murids	A species complex exhibiting considerable geographical genetic and biological variation. Apparently infective to humans primarily in E Asia and the Philippines
*P. westermani* (Kerbert, 1878): triploid worms	China, Japan, Korea	As above	As above	Disease caused by diploid and triploid worms is similar. But triploid individuals larger and reproduce by parthenogenesis
*P*. *skrjabini* (Chen, 1959) and subspecies such as *P*. *s*. *miyazakii*	E Asia (China, Japan), Thailand, extending to NE India	Pulmonary forms rare; pleural and ectopic forms usual	Canids, felids, mustelids, viverrids, murids, hystricids	A species complex. Humans not usually suitable definitive hosts
*P*. *heterotremus* Chen and Hsia, 1964	SW and southern China, Vietnam, Laos, Thailand, NE India	Usually pulmonary; ectopic cases reported	Felids, sciurids, murids	Possibly a species complex that includes a form described as *P. pseudoheterotremus* Waikagul, 2007
*P*. *africanus* Voelker and Vogel, 1965	W Africa: Equatorial Guinea, Cameroon, Nigeria, possibly Ivory Coast	Usually pulmonary, ectopic cases suspected	Lorids, cercopithecids, herpestids, viverrids	
*P*. *uterobilateralis* Voelker and Vogel, 1965	W Africa: Gabon, Cameroon, Nigeria, Liberia	Usually pulmonary, ectopic cases suspected	Canids, herpestids, mustelids, viverrids	
*P. gondwanensis* Bayssade-Dufour *et al*., 2014	Cameroon, Ivory Coast	Probably pulmonary	Viverrids, felids, canids	Little is known about this species. It awaits molecular characterization and studies on distribution
*P*. *kellicotti* Ward, 1908	N America: Mississippi Basin and Atlantic coast of the USA; Ontario and Quebec in Canada	Usually pleural and pulmonary (occasionally ectopic). Pleural symptoms may be severe	Didelphids, canids, felids, mustelids, procyonids, suids, bovids, murids	Rare in humans, but humans are permissive hosts
*P*. *mexicanus* Miyazaki and Ishii, 1968	Central and S America: Mexico, Costa Rica, Panama, Guatemala, Ecuador, Peru; probably other countries in the region	Usually pulmonary	Didelphids, cebids, canids, felids, mustelids, procyonids, suids	Possibly a species complex

Even among *Paragonimus* species that typically cause pulmonary disease, some worms may cause pleural disease, or stray beyond the thoracic cavity to cause ectopic paragonimiasis (e.g. Ahn *et al*., 2021), which can affect almost any part of the body. Sometimes, worms in ectopic sites produce eggs. Some species of *Paragonimus*, those in the *P. skrjabini* group, primarily cause ectopic disease (Table 1). Cerebral paragonimiasis is particularly dangerous, but cases have been reported involving the skin (often as migratory nodules), liver, abdominal organs and genitalia (reviewed in Blair, 2019). Cerebral paragonimiasis is the most frequently reported, presumably because it produces severe symptoms and can have devastating consequences (Amaro *et al*., 2016). In recent years there have been few reviews of ectopic paragonimiasis, but a number of interesting case reports have appeared (e.g. Wang *et al*., 2018; Al Bishawi *et al*., 2021; Paranjape *et al*., 2021).

Reports of misdiagnosed paragonimiasis are very common (e.g. Qian *et al*., 2021; Shu *et al*., 2021). Cao *et al*. (2020), in a literature survey, reported that misdiagnosis of paragonimiasis cases in China between 2009 and 2019 ranged between 69 and 89% (and see Ruan *et al*., 2019). Finding of eggs in sputum or faeces is the ‘gold standard’ for diagnosis. However, caution is needed in identifying eggs in faeces as those of *Paragonimus*: a number of unrelated trematodes that live in the intestine produce rather similar eggs. Sputum examination also has its limitations. Eggs often appear only intermittently in sputum in pulmonary cases (e.g. Ahn *et al*., 2021), and never in ectopic cases. To overcome these limitations, a range of immunodiagnostic approaches have been developed. Medical imaging is also valuable (e.g. Xia *et al*., 2016; Qin *et al*., 2019), but often yields images suggestive of other conditions. Further details on pathology, diagnosis and treatment will not be covered in this article. For information on these topics, see Blair (2019), Esteban *et al*. (2019), Wu *et al*. (2019), Yoshida *et al*. (2019) and Weissferdt (2020).

What follows is a list of questions, answers to which are becoming increasingly more necessary for a full understanding of the epidemiology of paragonimiasis and the evolution of members of the genus *Paragonimus*.

## Discussion and questions for future investigation

### Is the taxonomy of the genus *Paragonimus* now settled?

**Fig. 2.**
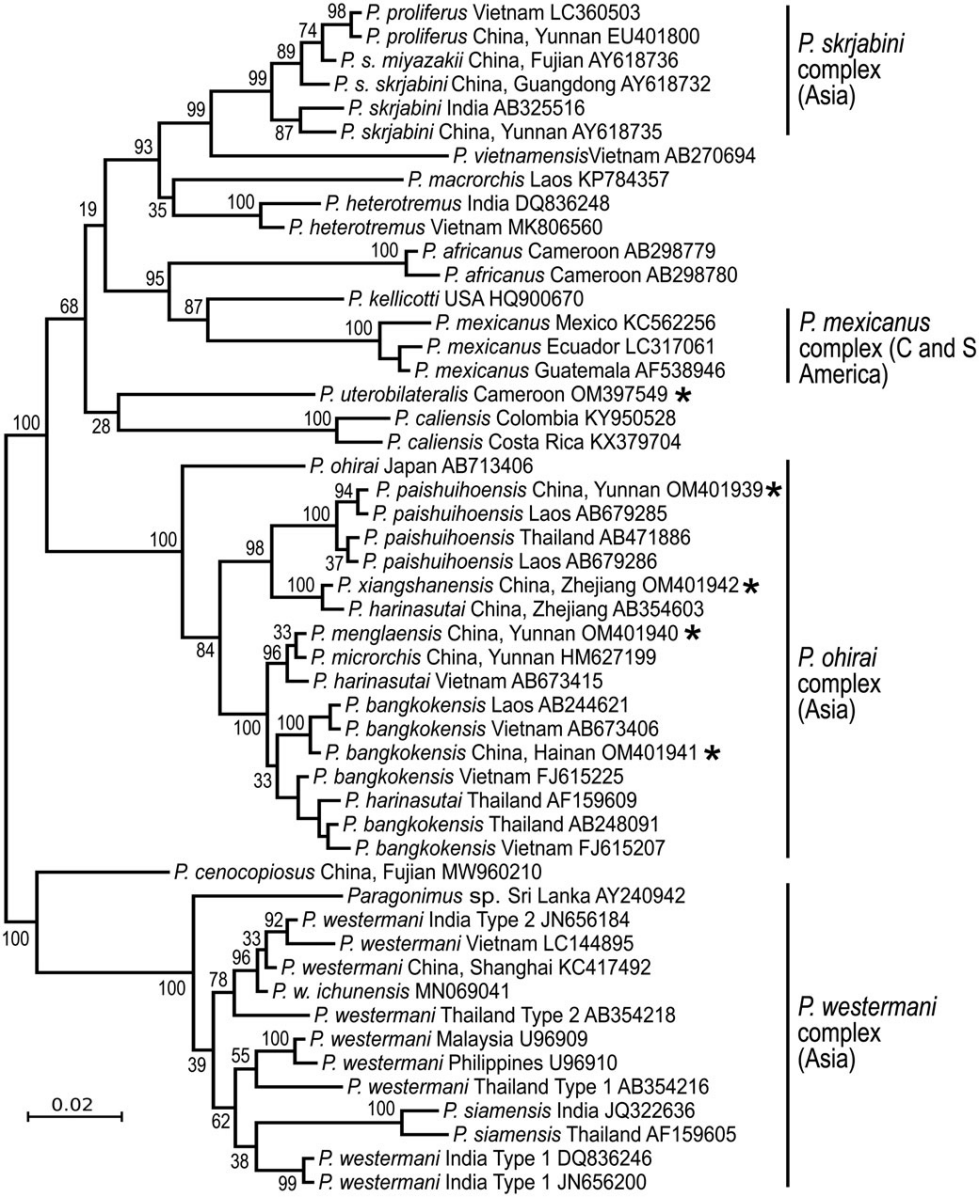
Phylogenetic relationships of selected groups of *Paragonimus* species, showing some of the species complexes within the genus. Midpoint-rooted Bayesian phylogenetic tree constructed in MrBayes v3.2.6 (Ronquist *et al*., 2012) and inferred using ITS2 sequences. The adjoining 5.8S and 28S gene sequences have been trimmed from the alignment before analysis, leaving only the spacer region (alignment length 291 bp). The optimum substitution model was K2P + G. Ten million generations were sampled every 2000. The first 25% of sampled trees were discarded as burn-in. Numbers at nodes indicate posterior probabilities. The name ‘*Paragonimus westermani*’ has been applied to sequences from most members of that complex for convenience. The tree includes five sequences not previously available in public databases (indicated with an asterisk), all obtained from metacercariae identified to nominal species on morphological grounds. Four of the unpublished sequences fall within the under-researched *P. ohirai* complex, all from China, and were originally included in analyses in Cui *et al*. (2003). Discussion relating to this complex can be found in Habe *et al*. (2013) and Yoshida *et al*. (2019).

*The answer is ‘No’*. Each continent has distinct species (Fig. 2), suggesting ancient origins of the genus *Paragonimus*. Blair *et al*. (1999) listed 50 nominal species in the genus (plus two in the genus *Euparagonimus* that should be referred to *Paragonimus*). Since then, three more species have been described from Asia (reviewed in Yoshida *et al*., 2019) and two from Cameroon in Africa (Bayssade-Dufour *et al*., 2014, 2015). Some of this multitude of species names must fall as synonyms. For example, Blair *et al*. (1999) suggested that seven nominal species were synonyms of *P. westermani*, and in 2005, Blair *et al*. suggested that at least another six nominal species were synonymous with *P. skrjabini*. Nevertheless, a large number of species do appear to be valid, especially in Asia and it is likely that more remain to be discovered, especially in Africa and South America. Miyazaki *et al*. (1980) mentioned the metacercaria of an unknown lung fluke in Mexico and Panama, and unidentified lung flukes have been reported from Brazil (Brenes *et al*., 1980). Amunárriz (1991) found distinctive, small metacercariae in Ecuador and coined the name *P. napensis* for these: nothing more is known about this form. Little (1968) commented on unidentified lung flukes in Central/South American mammals. All of this suggests that additional species will be found in the Americas, and of course, the earliest description of a lung fluke was of a worm from an otter in Brazil (Diesing, 1850), but the true identity of this parasite remains unknown and attempts to obtain more specimens have failed (discussed in Blair *et al*., 1999). In Africa, Appleton (2014) summarized reports of paragonimiasis in KwaZulu-Natal, South Africa, a region far from other known foci of the disease on the continent. The identity of the species is not known, nor is it clear whether it is endemic or has become established in South Africa following introduction of suitable hosts. Paragonimiasis might occur in baboons in Tanzania, another location far from known species (Bakuza, 2020). Rabone *et al*. (2021) have appealed for more research on the diversity and distribution of *Paragonimus* species in Africa. The two African species for which DNA sequence data are available do not appear to be closely related (Fig. 2).

Traditionally, identification of *Paragonimus* species has relied on morphological features of the adult worm (e.g. degree of lobation of testes and ovary, arrangement of spines on the body surface and relative sizes of oral and ventral suckers) and the form of the metacercaria (encysted or not in the crustacean host, number and thickness of cyst walls, diameter of cyst, form of the excretory bladder, etc.). These morphological features are reviewed in Blair *et al*. (1999) and Blair (2019). Unfortunately, there are too few morphological features of the adult stage for clear separation between the many nominal species, and features of the metacercarial stage are now known to be variable and unreliable for taxonomy (e.g. Blair *et al*., 2005; Devi *et al*., 2013; Doanh *et al*., 2016). In keeping with current fashion, molecular data in the form of DNA sequences have been brought into the fray. Sequences from three gene regions have been widely used: a portion of the mitochondrial *cox1* gene and of the nuclear ribosomal 28S rRNA gene, and the nuclear ribosomal internal transcribed spacer (ITS2). Molecular data have quickly demonstrated two opposing sets of findings. First, species that are morphologically similar over a large geographical region may actually consist of cryptic members of a species complex. Second, many nominal species, distinguished by their authors often on the basis of metacercarial morphology, should be lumped into a single species or species complex. Examples will be given below.

The best-known species, *P. westermani*, is really a complex of cryptic species that includes a second morphologically distinct species, *P. siamensis* Miyazaki & Wykoff, 1965. Adults of *P. westermani sensu lato* are nearly indistinguishable across the range. Human disease is caused by this species in Japan, eastern and northeastern China, the southern Far East of Russia, Korea and the Philippines. But surprisingly, human cases are not known from other parts of its range (e.g. Thailand, Malaysia and India, where there has been a single human case reported). This is not a consequence of local dietary habits in relation to freshwater crabs: *P. heterotremus* Chen & Hsia, 1964 infects residents of India and Thailand when they consume crabs. In addition to these and other biological differences (Blair, 2019), there are molecular differences among members of the *P. westermani* complex. A molecular phylogeny (Fig. 2) clearly shows the considerable diversity in ITS2 sequences across the range of the complex. Molecular data are lacking for some key regions within this range, in particular Myanmar. Possibly, sequences from additional locations would find evidence of clinal variation across the range, rather than the distinct clusters noted in the figure. It is remarkable that two forms of *P. westermani*, differing considerably in their molecular sequences but not their adult morphology, can be found in the same region of NE India (Devi *et al*., 2013), and another two in Thailand (Binchai *et al*., 2007). One of the forms in India has ITS2 sequences similar to those in China/Japan/Korea (Fig. 2). There is as yet no clear explanation for this. But nomenclatural tangles await those who seek to apply species names, new or existing, to the different forms.

*Paragonimus* species from Latin America provide further examples of taxonomic difficulties and the fact that molecular data can add another layer of complexity rather than taxonomic resolution. Since the mid-20th century, six species have been named from South and Central America, the first being *P. mexicanus* Miyazaki and Ishii, 1968, described from Colima, Mexico. Tongu (2001) was of the opinion that all six species represented *P. mexicanus*, whereas Miyazaki *et al*. (1980) believed that only *P. ecuadoriensis* Voelker and Arzube, 1979 and *P. peruvianus* Miyazaki *et al*., 1969 should be regarded as synonyms of *P. mexicanus*. Early molecular work (Iwagami *et al*., 2003) found only a single base difference between ITS2 sequences of worms (putative *P. mexicanus*) from Ecuador and Guatemala, suggesting that they were conspecific. However, partial *cox1* sequences did indicate a geographical distinction between these two countries. López-Caballero *et al*. (2013) sequenced additional putative *P. mexicanus* from three locations in Mexico including the type locality. They recognized three morphotypes, each from a different location. The morphotypes differed in degree of lobation of the gonads in adults and the pattern of papillae on the ventral sucker of the metacercariae. ITS2 sequences did not convincingly segregate according to morphotype, but *cox1* sequences did so segregate. Taken together, the data were regarded as evidence of three distinct species in Mexico (one being *P. mexicanus*), all distinct from the Ecuadorian form, which would then retain the name *P. ecuadoriensis*. Thus, the idea that *P. mexicanus* is a species complex was born. Landaverde-González *et al*. (2022) added *cox1* sequences from a river system in Guatemala and incorporated other published data from Costa Rica. By application of species-delimitation software, they inferred the existence of the same three species in Mexico (plus one in Ecuador) reported by López-Caballero *et al*. (2013), plus an additional species in Costa Rica. This supported the proposal that *P. mexicanus* is a complex of several cryptic species. Given that this taxon is thought to be distributed from Mexico to Peru (and Venezuela; Díaz *et al*., 2010), and that molecular data are not available from much of this range, a great deal of further work awaits researchers. Such work will need to determine locations of geographical boundaries between putative cryptic species, the extent to which morphotypes map to molecular groupings and whether some of the geographical molecular variation can be attributed to clinal variation. With respect to the second of these, Hernández-Chea *et al*. (2017) found three morphotypes (differing in arrangement of papillae on the ventral sucker) among metacercariae of *P. mexicanus* in Costa Rica: these morphotypes did not segregate cleanly on phylogenetic trees based on *cox*1, 28S or ITS2 sequences.

The status of another species in the region, *P. caliensis* Little, 1968, seems to have been settled by the use of molecular data (reviewed in Hernández-Chea *et al*., 2017). This species was originally described from Colombia (Little, 1968). Tongu (2001) and Vélez *et al*. (2009) regarded this species as a variant of *P. mexicanus*. Hernández-Chea *et al*. (2017) found a small number of metacercariae in Costa Rica that were consistent with the description of those of *P. caliensis* from Colombia. Molecular data demonstrated that these metacercariae did indeed belong to a very distinct branch of the genus (Fig. 2). Hernández-Chea *et al*. (2017) noted two different patterns of papillae around the ventral sucker in these metacercariae, but sequences from the different types intermingled in phylogenetic trees. Lenis *et al*. (2018) added morphological and molecular data from Colombia, including the type locality for *P. caliensis*. They also found some morphological variation that was not correlated with a molecular phylogeny. The full geographical range of *P. caliensis* remains unknown. Nor is it known whether the species can infect humans. Lenis *et al*. (2018) noted that it can be difficult to distinguish *P. caliensis* from *P. mexicanus*, but that molecular data should be used for this purpose.

There are more examples of the complexities of interpreting molecular and morphological data in understanding the nature of lung-fluke species or species complexes. A brief discussion of this in relation to *P. heterotremus* can be found in Saijuntha *et al*. (2021).

### Are all details of the life cycle clear?

*The answer is ‘No’*. There have been some excellent studies on experimental completion of life cycles of some populations of *Paragonimus* species in Japan (reviewed in Yokogawa *et al*., 1960) and the Americas (e.g. Ameel, 1934; Brenes *et al*., 1980). But many other life cycles have been inferred from limited experimental work and/or untested assumptions. Trematode species tend to be very specific for their snail hosts (Blair *et al*., 2001). Knowledge of the habitat of the snail host can provide clues to epidemiology and possible transmission sites. For example, in Vietnam, members of the *P. skrjabini* complex typically use locally endemic tiny rissoidean snails living in small fast-flowing mountain streams (Doanh *et al*., 2018*b*), whereas members of the *P. westermani* complex use larger snails (family Pachychilidae) typically found in larger streams. The snail hosts remain unproven for all African species of *Paragonimus*, although some candidate species have been suggested (Cumberlidge *et al*., 2018). Analysis of life cycles is made difficult by the shifting taxonomy of snails and of crabs, as well as outright misidentification. In particular, there is a tendency for researchers to assume that all microcercous cercariae found in snails are those of *Paragonimus* species, but this type of cercaria is known from other trematode families (especially Troglotrematidae), which may even co-occur with *Paragonimus* in the same snail species (Doanh *et al*., 2020). Molecular data have helped to untangle these problems (Doanh *et al*., 2018*b*, 2020) and need to be applied more widely in lieu of experimental infections of snails.

### Is opportunistic use of paratenic hosts a feature of the life cycle of all *Paragonimus* species?

This phenomenon is best known for *P. heterotremus* (e.g. Voronova *et al*., 2020) and *P. westermani* (e.g. Banzai *et al*., 2021). Persistence of larval *P. westermani* in inhabitants of the Russian Far East may indicate that humans can also be potential paratenic hosts (Belov *et al*., 2021). Use of paratenic hosts has also been reported for *P. mexicanus*, *P. kellicotti* Ward, 1908 and the *P. skrjabini* complex (Blair *et al*., 1999). It remains to be demonstrated whether other species can do this.

### Is the prevalence of paragonimiasis decreasing?

*The answer is a qualified ‘Yes’*. Factors that are likely to reduce incidence include mass drug treatment, increased quality control of marketed foods, public awareness of the disease and how to avoid it, economic development removing increasing numbers of people from natural environments, changing lifestyles and diets, and the effects of habitat degradation and pollution reducing numbers of natural hosts (more details can be found in Blair, 2019; Hong and Yong, 2020; Song *et al*., 2021; Zhou *et al*., 2021). Factors that might tend towards increased infection rates could include an increase in affluent urban dwellers buying and consuming freshwater crustaceans (‘urban paragonimiasis’), or its polar opposite, increased inequalities and poverty forcing people to forage for wild foods (Hotez, 2017). These opposing economic and social forces presumably prompted Zhou (2022) to identify paragonimiasis as both an ancient and a re-emerging disease. Land-use change (Chakraborty *et al*., 2019) and some forms of environmental protection (Zhou *et al*., 2021) might also tend to cause increases, as would increasing interactions of rural dwellers with the natural environment. Culinary habits may over-ride knowledge, even when people are aware of the risks of eating under-cooked food (Chaisiri *et al*., 2019), thus perpetuating preventable infection. Even more extreme is the repudiation of science in many countries, placing the health of many at risk despite efforts of educators (Hotez, 2017).

Paragonimiasis has all but disappeared from South Korea, Japan and the island of Taiwan. In Japan, some cases are reported every year in hunters who have eaten raw meat of pigs or deer: re-emerging paragonimiasis (Banzai *et al*., 2021). Prevalence in China has dropped considerably. Qian *et al*. (2019) have cited results from two national surveys 10 years apart. The first of these (2001–2004) used serological methods and showed an overall prevalence of 1.7%. The second survey used faecal examination and estimated a prevalence of 0.005%. Given the different methods used, and the likely overestimation from serology, it is hard to draw firm conclusions, but all the indications are of a great reduction in China. However, worldwide numbers of people infected with paragonimiasis, published by the Global Burden of Disease study (2016), do not indicate a substantial decline during the early 21st century (Table 2).

**Table 2.** Estimates of global numbers of cases of paragonimiasis, stratified by severity

	Prevalence in thousands (95% CI)	
Severity	Year 2005	Year 2015
Asymptomatic paragonimiasis	23 333 (13 242–30 252)	22 772 (12 991–29 225)
Mild paragonimiasis	4861 (677–11 657)	4754 (646–11 395)
Moderate paragonimiasis	1141 (156–2732)	1114 (150–2643)
Severe paragonimiasis	1383 (194–3270)	1351 (189–3208)
Cerebral paragonimiasis	222 (64–450)	182 (59–353)

Extracted from the Global Burden of Disease 2015 study, Supplement 2 page 10.

Note the wide confidence intervals in Table 2. There are several reasons why prevalence of paragonimiasis is difficult to estimate. First, infected individuals may be asymptomatic (e.g. Kusolsuk *et al*., 2020) or mildly symptomatic, failing to perceive that they have a parasitic infection. Second, diagnosis can be difficult unless the clinicians have reasons to suspect paragonimiasis. This leads to probable under-reporting of cases. Eggs are often not present in sputum, even in pulmonary cases, and are not present in ectopic cases. There are numerous reports of sufferers being misdiagnosed with tuberculosis and enduring lengthy and ineffective treatments for that disease (see above and examples from the Philippines cited by delos Trinos *et al.* 2020). Third, diagnostic methods can differ dramatically in the prevalence estimates they support. Immunodiagnostic methods, increasingly preferred because of the difficulties in parasitological diagnosis, can yield positive results in a remarkably high proportion of a tested population (e.g. Qian *et al*., 2019), which might reflect current or past infection or be due to cross-reactions with other parasites, or to use of inappropriate cut-off values.

### Has the genomic era had an impact on the study of paragonimiasis?

*The answer is ‘Yes’*, but we are still very far from having genomic/genetic data to explain, for example, why some species or populations of lung flukes can infect humans and others cannot.

Since the publication of an earlier review on gene diversity and genetic variation in *Paragonimus* (Blair *et al*., 2016), genomes and/or transcriptomes of several *Paragonimus* species have been published (Li *et al*., 2016; Oey *et al*., 2019; Li *et al*., 2020; Rosa *et al*., 2020). In some respects, the datasets generated by these studies revealed findings that were expected: genes of *Paragonimus* species have many homologues in other foodborne trematodes (Oey *et al*., 2019). However, analysis of genomic data from several species/populations of *Paragonimus* permits identification of genes that are conserved and unique to the group. This provides an indication of genomic features likely associated with the shared habitat of that group. In the case of *Paragonimus* species, such genes include those that probably function in host-tissue invasion, immune modulation and possibly in digestion of host blood (Rosa *et al*., 2020). Genomic studies also make it possible to identify expanded or contracted gene families, providing other clues to biological mechanisms. Another major use of genomic data is to identify complete genes coding for e.g. potential diagnostic antigens and drug targets, which have been partially or completely characterized by other methods (Li *et al*., 2016; Rosa *et al*., 2020; Curtis *et al*., 2021). Genomic studies generate huge volumes of information. Much of this cannot yet be placed within its biological context. Bioinformatic analyses can facilitate extraction of useful information, exemplified by Li *et al*. (2021*a*), who attempted to identify virulence factors in lung flukes. The mass of data acquired to date will be reference material for future work, allowing refinement of interpretation, development of more-informed hypotheses and experimental studies into the foreseeable future.

*Paragonimus* species have complex life cycles, during which they utilize hosts of three unrelated taxa (molluscs, crustaceans and mammals). Transcriptomic data from a single life stage cannot reveal much about how the parasite survives in hosts of other life stages. All studies to date have used stages from mammals, albeit at different developmental points in some cases. For example, Rosa *et al*. (2020) obtained transcriptomic data from both adult and pre-adult worms in mammals. Perhaps not surprisingly, they found that eggshell proteins were less expressed in young adults than in fully mature worms. Similarly, Li *et al*. (2021*b*) used *Paragonimus proliferus* Hsia & Chen, 1964 (a member of the *P. skrjabini* complex: see Fig. 2) at different stages of maturity in rat lungs and demonstrated changes in gene expression through time. Given the small size (and therefore lower yield of nucleic acids) of miracidia, intra-molluscan stages, cercariae and even metacercariae, completing transcriptomic analysis of each of these stages will be challenging.

Genomic data may be able to tell us more about diversity within a species complex. Rosa *et al*. (2020) compared their genome data from a Japanese form of *P. westermani* with the genome of an Indian member of the complex. At the nucleotide level, they found a mean sequence identity of 87.6%. This seems rather low for supposed conspecifics!

### Is climate and global change affecting incidence and distribution of paragonimiasis?

*The answer is ‘I don't know’*. The effect of climate change on distribution and prevalence of parasites is a popular topic (e.g. Utaaker and Robertson, 2015; Carlson *et al*., 2017; Booth, 2018). But it needs to be considered alongside a separate anthropogenic phenomenon: the translocation of species that become invasive. In a review considering both of these aspects, Pozio (2020) downplayed the idea that *Paragonimus* species would spread because of the requirement for specific hosts to complete the life cycle. As mentioned earlier, it is likely that *Paragonimus* species are highly specific for their snail hosts. The apparent broad specificity, noted for some *Paragonimus* species by Blair *et al*. (1999), is probably an illusion, due to frequent incorrect identification of snails, and the presence of cryptic species of *Paragonimus*, each of which might be very specific (Blair *et al*., 1999). The degree of specificity for the crustacean host is not well known but might be broader than for snails (e.g. see Zhou *et al*., 2021 for a list of reported crustacean hosts in China). Substantial changes in geographical distribution of known host snails have not been reported. However, a cautionary note is required: one freshwater gastropod, *Melanoides tuberculata* (Müller, 1774) has now become widely established in suitable habitats around the world (e.g. Nguyen *et al*., 2021). This species has been listed as a host for *Paragonimus* species, but this has been questioned (e.g. Blair *et al*., 1999; Doanh *et al*., 2013). It is the case, however, that some trematodes have started to exploit *M. tuberculata* in regions where it is invasive (e.g. Tolley-Jordan *et al*., 2022). This could be due to translocation of a trematode from the source range of the snail. But alternatively, it could be a consequence of host switching by local trematodes to exploit *M. tuberculata*: in such a way, parasites may hitch-hike with a newly acquired invasive snail host to other parts of the world. Crustaceans, too, can be invasive, bringing with them the danger of introductions of new parasites. At present, the only known crustacean host for *Paragonimus* that has become invasive is the estuarine Chinese mitten crab (Blair *et al*., 1999). In its home range (China and Korea), this crab is a host for *P. westermani*. The crayfish *Procambarus clarkii* (Girard, 1852) has been introduced to Japan and many parts of China. It is known to be a potential intermediate host for *P. westermani* (see Feng *et al*., 2018; Zhu *et al*., 2019).

## Concluding remarks

Members of the genus *Paragonimus* receive rather little attention from those interested in the neglected tropical diseases. However, they offer much of interest for researchers studying their evolution, taxonomy and adaptations to their parasitic way of life. Even if the incidence of human infections is reduced, lung flukes will continue to cycle through their natural sylvatic hosts and are unlikely to vanish from view. Future work on these as model organisms, using methods old and new, will throw light on their way of life that will likely also apply to other foodborne trematodes.
